# Tizanidine-Induced Bradycardia Without Concomitant Medications: A Case Report

**DOI:** 10.7759/cureus.60581

**Published:** 2024-05-19

**Authors:** Kensuke Kikuchi, Kei Tsukamoto, Haruka Kikuchi, Takashi Saito, Fumiaki Mori

**Affiliations:** 1 Cardiology, Yokohama Medical Center, Yokohama, JPN

**Keywords:** a case report, bradycardia, cyp1a2, cytochrome p450 cyp1a2, drug-induced bradycardia, tizanidine

## Abstract

A 37-year-old woman was admitted to our hospital due to a loss of consciousness. She had been taking 2 mg of tizanidine for two months to manage shoulder muscle pain at night. On admission, an electrocardiogram showed sinus bradycardia with a heart rate of 30 bpm and QT prolongation (QTc 495 msec). She had a temporary pacemaker inserted in the catheterization room, after which an improvement in her level of consciousness was observed. There were no apparent endocrine disorders or structural heart diseases. The administration was discontinued after admission, and 12 hours after admission, her heart rate normalized to a sinus rhythm of 70-100 bpm, and QTc improved to 431 msec. Therefore, she was diagnosed with tizanidine-induced bradycardia. Although reports of tizanidine-induced bradycardia are rare, tizanidine's central α2 agonistic effects can cause bradycardia, necessitating caution.

## Introduction

Tizanidine is an adrenergic α2 receptor agonist that acts on the locus coeruleus in the pons to inhibit neurotransmission responsible for muscle tension, thereby alleviating excessive muscle tone [1]. It is commonly prescribed for disorders such as cervicobrachial syndrome, back pain, spastic paralysis, migraines, and tension headaches. Known side effects of tizanidine include drowsiness, dry mouth, weakness or fatigue, stomach discomfort, rash, and itching [2], with bradycardia being a rare occurrence. Tizanidine is primarily metabolized in the liver by cytochrome P450 (CYP) 1A2 [3]. Concurrent use with CYP1A2 inhibitors may induce side effects even at standard dosages, and several cases of drug-induced bradycardia have been reported with concomitant use [4-7]. We report a case of tizanidine-induced bradycardia without any evident drug interactions.

## Case presentation

**Figure 1:**
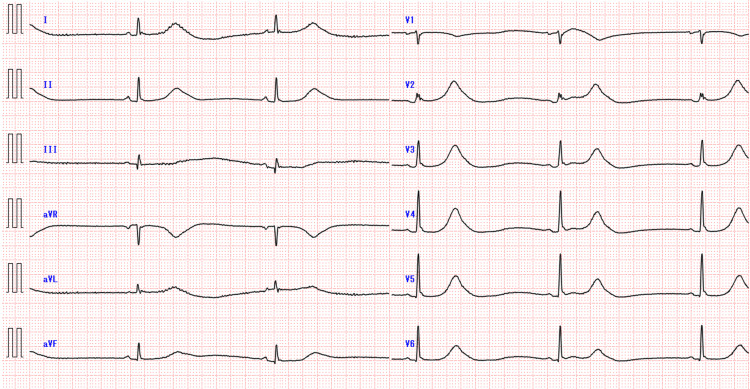
Electrocardiogram showing sinus bradycardia with prolonged QTc interval

A 37-year-old woman was brought to the emergency room due to loss of consciousness while commuting to work. Upon arrival at the hospital, her blood pressure was 98/61 and her pulse rate was 30 bpm. Her oxygen saturation was normal, and she had consciousness impairment with a Glasgow Coma Scale score of E3V4M6. She had a history of bipolar disorder and epilepsy. Her medications included levetiracetam 200 mg, lamotrigine 75 mg, zolpidem 10 mg, and flunitrazepam 2 mg (as needed for insomnia). She had been taking tizanidine 2 mg before sleep for nocturnal muscle tension for two months and had been aware of daytime dizziness since the same time. The last dose of tizanidine was taken 12 hours before arrival. She had no family history of cardiac disease or smoking history. An electrocardiogram revealed sinus bradycardia with a heart rate of 30 beats per minute and a prolonged QTc interval of 495 ms (Figure 1).

**Figure 2:**
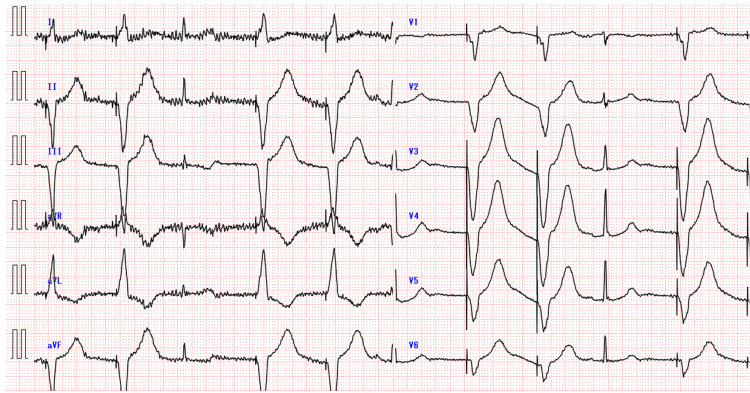
Electrocardiogram after insertion of a temporary pacemaker (activated in VVI mode at 60 bpm) VVI: pacing in the ventricle; sensing in the ventricle; inhibit

Echocardiography showed preserved left ventricular function and no apparent organic abnormalities. Laboratory testing revealed elevated BNP at 321 pg/ml. The blood glucose level was 106 mg/dL. Troponin I and CK-MB were negative. Thyroid function and electrolytes were also normal. A chest X-ray revealed no abnormal findings. CT showed no cerebral hemorrhage. According to her husband, she was adhering to her medication regimen and had no previous medication-related problems. A temporary pacemaker was placed and activated in VVI mode (pacing in the ventricle; sensing in the ventricle; inhibit) at 60 bpm, resulting in immediate improvement in her level of consciousness (Figure 2).

**Figure 3:**
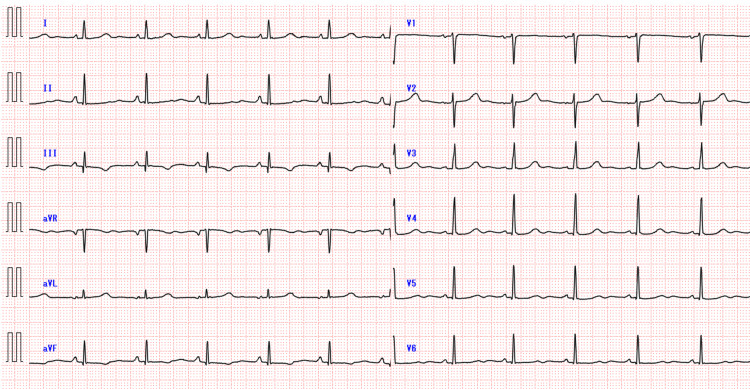
Electrocardiogram on the second day of admission

After admission, the administration of tizanidine was discontinued while other medications were continued. Twelve hours after admission (24 hours after the last dose of tizanidine), her heart rate improved to a sinus rhythm of 70-100 bpm and her QTc improved to 431 ms (Figure 3). 

No further episodes of bradycardia were observed thereafter, and on the second day of admission, the temporary pacemaker was removed, and she was discharged on the fifth day. After discussing with her, we decided not to insert a permanent pacemaker. A 24-hour Holter electrocardiographic monitoring performed one month after discharge showed no bradycardia or pauses.

## Discussion

As a differential diagnosis for bradycardia, various conditions need consideration such as hypothyroidism, hypothermia, increased intracranial pressure, sick sinus syndrome, carotid sinus hypersensitivity, structural heart disease, electrolyte abnormalities, and drug-induced bradycardia due to beta-blocker therapy. In this patient, the above diseases were ruled out, tizanidine was discontinued, and the heart rate returned to normal levels within 24 hours. Therefore, she was diagnosed with tizanidine-induced bradycardia. Also, the observed QT prolongation was considered to be due to tizanidine-induced bradycardia, because the likelihood of QT prolongation due to concomitant medications was low, except in the case of high doses of zolpidem. [8-10].

Tizanidine, a centrally acting α2 agonist, is prescribed for conditions involving muscle relaxation, such as multiple sclerosis [1]. The primary mechanism of action of α2 agonists is to stimulate presynaptic α2 receptors in the central nervous system, activating inhibitory neurons and reducing sympathetic output via a negative feedback mechanism. This results in an overall decrease in the secretion of the catecholamine, norepinephrine, causing a decrease in blood pressure and heart rate [11,12].

Maximum plasma concentrations are typically achieved 0.75-2 hours after tizanidine administration. Tizanidine is metabolized in the liver by the cytochrome P450 isozyme CYP1A2. The elimination half-life ranges from 2.1 to 4.2 hours in patients with normal renal function [2]. However, there are large individual differences in CYP1A2 activity: among the CYP1A2 gene polymorphisms, higher enzyme activity was observed in homozygotes or heterozygotes for the -163C>A polymorphism (rs762551) compared to wild type [13]. Tobacco also induces CYP1A2, which can cause adverse effects after abrupt smoking cessation [14]. CYP1A2 inhibitors may inhibit tizanidine metabolism and increase serum concentration [3].

Commonly reported side effects of tizanidine include dry mouth, drowsiness, asthenia, bradycardia, dizziness, hallucinations, gastrointestinal disturbances, and elevated liver transaminases [2]. Reports of bradycardia induced by tizanidine at usual doses are very few in PubMed searches. A few cases of tizanidine-induced bradycardia with concomitant use of CYP1A2 inhibitors have been reported: one case each with loxoprofen [4], ticlopidine [5], lisinopril [6], and rofecoxib [7]. A patient with tizanidine-induced bradycardia in combination with loxoprofen had an irreversible, permanent pacemaker implanted. In this case, because the bradycardia was reversible, a permanent pacemaker was not implanted.

There are fewer reported cases of bradycardia with tizanidine alone without obvious concurrent medications. Cortes et al. reported bradycardia in a 93-year-old woman after the initial administration of tizanidine [15]. Although there were no obvious concurrent medications in this case, the patient had a history of atrial fibrillation.

Masood et al. reported tizanidine-induced bradycardia in a patient with concomitant adrenal insufficiency [16]. The patient was taking a relatively high dose of tizanidine (12 mg).

In this case, the patient experienced tizanidine-induced bradycardia at a low dose of tizanidine (2 mg) without drug interactions, metabolic disorders, or structural cardiac disease, and there was no history of sudden smoking cessation that could cause a rapid decrease in CYP1A2 enzyme activity.

## Conclusions

We report the case of a 37-year-old woman with tizanidine-induced bradycardia without concomitant CYP1A2 inhibitors and metabolic abnormalities or structural heart disease. Although drug-induced sinus bradycardia caused by tizanidine is rarely reported, tizanidine has a central α2 stimulating effect and may cause bradycardia. Drug-induced bradycardia should be considered when starting tizanidine, even in the absence of comorbidities, structural cardiac disease, or concomitant use of CYP1A2 inhibitors.
